# Adjustment Method of College Students' Mental Health Based on Data Analysis Under the Background of Positive Psychology

**DOI:** 10.3389/fpsyg.2022.921621

**Published:** 2022-06-30

**Authors:** Sibo Yang, Lin Lin, Xue Zhang

**Affiliations:** ^1^School of Education, Harbin Normal University, Harbin, China; ^2^School of Marxism, Jinzhou Medical University, Jinzhou, China

**Keywords:** positive psychology, virtual reality, mental health of college students, psychological counseling, health problems, Apriori algorithm

## Abstract

Colleges and universities are in an important position to train builders and successors of the socialist cause whilst promoting quality education. Mental health education is an important foundation and condition for comprehensively improving students' overall quality. This research explores adjustment methods for college students' mental health based on virtual reality under the background of positive psychology. It discusses the importance of system requirements analysis in the software development process, analyzes the system's functional requirements, safety requirements, and software and hardware requirements, and uses the Apriori algorithm to explore the influencing factors of college students' mental health. Based on the system engineering method and using the data mining clustering method undertake detailed analysis and research on the mental health of college students, it then designs an anomaly mining algorithm based on clustering to quickly find anomalous data health problems. The interface design of the system is concise and the operation is simple. Users can conveniently input, query, and count information according to the various controls on the interface, which fully embodies human-oriented characteristics. Exploration of the characteristics of students' frequent Internet access ensures the efficiency, accuracy, and comprehensiveness of the evaluation and consultation work, facilitating psychological counseling for teachers and students, and saving paper. By establishing a data mining model, mining the database, and learning about different student groups and their respective characteristics, we discuss our research on student psychology and summarize the mental health status and gender, adaptation and anxiety, introversion, emotionality, and calmness of college students. We also consider the relationship between sex, negative, and courage. Using positive psychology theory, we examine the positive experiences of students and interconnected qualities, to build a mental health practice system. In the experiment, the happiness index evaluation of the virtual reality treatment system group was significant, *P* = 0.002 < 0.05. Mental health education plays an important role in cultivating the healthy psychology of college students, developing their psychological potential, enhancing their adaptability, and improving their personality. This analysis based on actual data provides a reliable basis for psychological educators to improve the efficiency and effectiveness of school psychological counseling and to facilitate schools in establishing new methods of early prevention and intervention for psychological disorders, enabling institutions to create a healthy atmosphere for college students.

## Introduction

The physical, physical, and psychological relationships of college students are delicate and need to be coordinated. If their mental state is not well adjusted, they are prone to psychological problems, which can affect their studies, life, and other aspects. Teachers can guide them to develop in a healthy and benign direction. It is therefore important that teachers understand and treat the psychological conditions of students in university.

Constructivism is a theory about knowledge and learning, emphasizing the initiative of learners, and believes that learning is a process in which learners generate meaning and construct understanding based on original knowledge and experience, and this process is often completed in social and cultural interactions. The modern network education environment provides the necessary means for the implementation of constructivist education. A networked mental health supplementary education system adapts to the requirements of constructivist learning theory and provides students with a network resource platform for autonomous learning, active construction of knowledge, and collaborative communication (Romero et al., 2021). The present paper describes the construction and application of an online psychological health education system for college students, a new field that also embodies the constructivist learning model. Improving the mental health level of college students is an important guarantee for college students to move toward modernization, the world, and the future. The development of students is reflected in their attitude and perspective of the world, ability to love people around them, their ability to work, and the wisdom and emotional factors embodied in all aspects of life, which guide college students to live an active lifestyle.

This study uses cluster analysis, which can be used either as a stand-alone algorithm or as a preprocessing step with other data mining algorithms and is an important research topic in the field of data mining. The basic idea of cluster analysis is to classify samples according to the principle of “clustering together.” The clustering analysis method is an unsupervised learning process that aggregates things into classes according to some attributes so that the similarity between different classes is as small as possible, and the similarity between the same classes is as large as possible, to realize the classification of data.

This article developed computer networks to undertake mental health tests and analyses. Students are receptive to communicating with computers, and this interaction can reflect their mental state more truthfully. Test results are also able to be completed and processed faster than paper answers. This test method also allows teachers to identify problems early so that they can quickly come up with solutions. Furthermore, paper can be saved, conserving resources, and reducing environmental pollution. We used cluster mining on the mental health database, to find out different student groups and their respective characteristics, to research student psychology, and actively guide students to have healthy mental states, enabling them to study and live, which has great significance.

## Related Work

In order to improve and optimize the decision-making of affairs and improve the efficiency of the school's psychological counseling for college students, it is necessary to make an accurate analysis of this information in a timely and accurate manner. Evans-Lacko believes that the treatment gap between the number of people with mental disorders and the number of people receiving treatment is a major public health challenge (Evans-Lacko et al., 2017). Leese believes that measuring the impact of different types of service provision on the opinions of service users is important for planning mental health services (Leese et al., 2018). Askari studied the impact of training on communication and conflict resolution skills on the mental health of Iranian couples based on the PREPARE/ENRICH plan (Askari et al., 2017). Freeman believes that mental health issues are inseparable from the environment (Freeman et al., 2017). Emotion is the collective term for a series of self-emotional expressions of human beings. It is the normal physiological and psychological state of objective things produced under subjective cognitive experience. Normal emotion production and expression are important ways and means for human beings to adapt to survival and communicate with each other. Factors such as neuropathy, external pressure, or strong stimuli may cause involuntary emotional fluctuations or changes in emotional nature, which can cause emotional disorders in severe cases. The main clinical manifestations of affective disorders include emotional instability, mania, and depression, among which depression is one of the representative diseases of mental illness. Recent research has started to focus on patients with depression, which involve the highest fatalities due to relapses and high prevalence. Traditional college students' mental health education information knowledge is mostly carried in limited written textbooks and stored in the library.

## Mental Health Adjustment Methods of College Students Based on Virtual Reality

### The Construction of College Students' Psychological Correlation Analysis System

Psychological problems cause problems for college students and can cause harm. Most colleges and universities simply strengthen theoretical education and management. To better carry out certain targeted guidance and education on the psychology of college students, this paper used the association rule mining algorithm in data mining, namely the Apriori algorithm. We selected some psychological factors, obtained part of the data from the college entrance psychological evaluation system, and then mined the collected data to find practical rules. This paper makes a reasonable analysis of the irregular spatial distribution and the global optimal solution. By testing the experimental data, data mining technology was applied to the analysis of the mental health of college students.

In the article, we define *x*_1_ as gender, *x*_2_ means major, *x*_3_ is grade.. and *x*_n_ refers to mental illness. The sample information of a college student is then recorded as an n-dimensional coordinate point, and all the data information is the set of A (Levecque et al., 2017).


(1) $$\begin{array}{l} A=\left(x_{1},x_{2},x_{3},x_{4},\ldots Kx_{n}\right) \end{array}$$

Let *x*_2_ = *mj*1, we can get the survey data set f (Chekroud et al., 2017) of college students majoring in science and engineering.


(2) $$\begin{array}{l} f=\left\{A|x_{2}=mj1\right\} \end{array}$$

For example, if *x*_2_ and x_3_ can be set at the same time, we can get the survey data f_1_ of lower grade science and engineering majors.


(3) $$\begin{array}{l} {\text{f}}_{1}=\underset{i}{\sum }\left(A_{i}|x_{2}=mj1\cap x_{3}=gd0\right) \end{array}$$

It is again restricted to *x*_3_, and survey data of lower-grade science and engineering majors can also be obtained (Zhang and Chen, 2017).


(4) $$\begin{array}{l} {\text{f}}_{2}=\underset{i}{\sum }\left(A_{i}|x_{2}=\text{f}\cap x_{3}=gd0\right) \end{array}$$

(5) $$\begin{array}{l} S\;(D)=-\overset{m}{\underset{i=1}{\sum }}p\log\;(p) \end{array}$$

The expected information needed to classify the sample on the classification of attribute A can be obtained by the following formula (Thornicroft and Semrau, 2018; Deepak et al., 2022):


(6) $$\begin{array}{l} S_{A}\;(D)=\overset{v}{\underset{j=1}{\sum }}\frac{\left|D_{i}\right|}{\left|D_{j}\right|}\times S\;(D) \end{array}$$

Where $\overset{v}{\underset{j=1}{\sum }}\frac{\left|D_{i}\right|}{\left|D_{j}\right|}$ is the weight of the subset.

Since the value of attribute A is known, the entropy is reduced. This entropy reduction can be obtained by the following formula (Eisenberg et al., 2018):


(7) $$\begin{array}{l} G\;(A)=S\;(D)-S_{A}\;(D) \end{array}$$

The law of total probability knows:


(8) $$\begin{array}{l} P\;(x)=\overset{n}{\underset{i=0}{\sum }}P\;(b|A)P\;(A) \end{array}$$

### Psychotherapy System

Data mining refers to the process of searching for information hidden in a large amount of data through algorithms. Data mining is generally related to computer science and achieves these goals through a number of methods such as statistics, online analytical processing, intelligence retrieval, machine learning, expert systems (relying on past rules of thumb), and pattern recognition. To realize a psychological crisis prevention system that integrates three data mining methods to complement each other's strengths, the system has the following performance requirements: smooth transmission of information between various modules, smooth interaction of data between various models, fast system response, friendly user interaction interface, and easy operation. The embedded data mining module has good compatibility with the original system, smooth data exchange, and high requirements for self-maintenance and reliability of the embedded module. It can achieve a more complete analysis and meet the mutual comparison, correct and modify the calculation results of data mining models suitable for different occasions, and nest other models in one of the data mining models.

**Figure 1:**
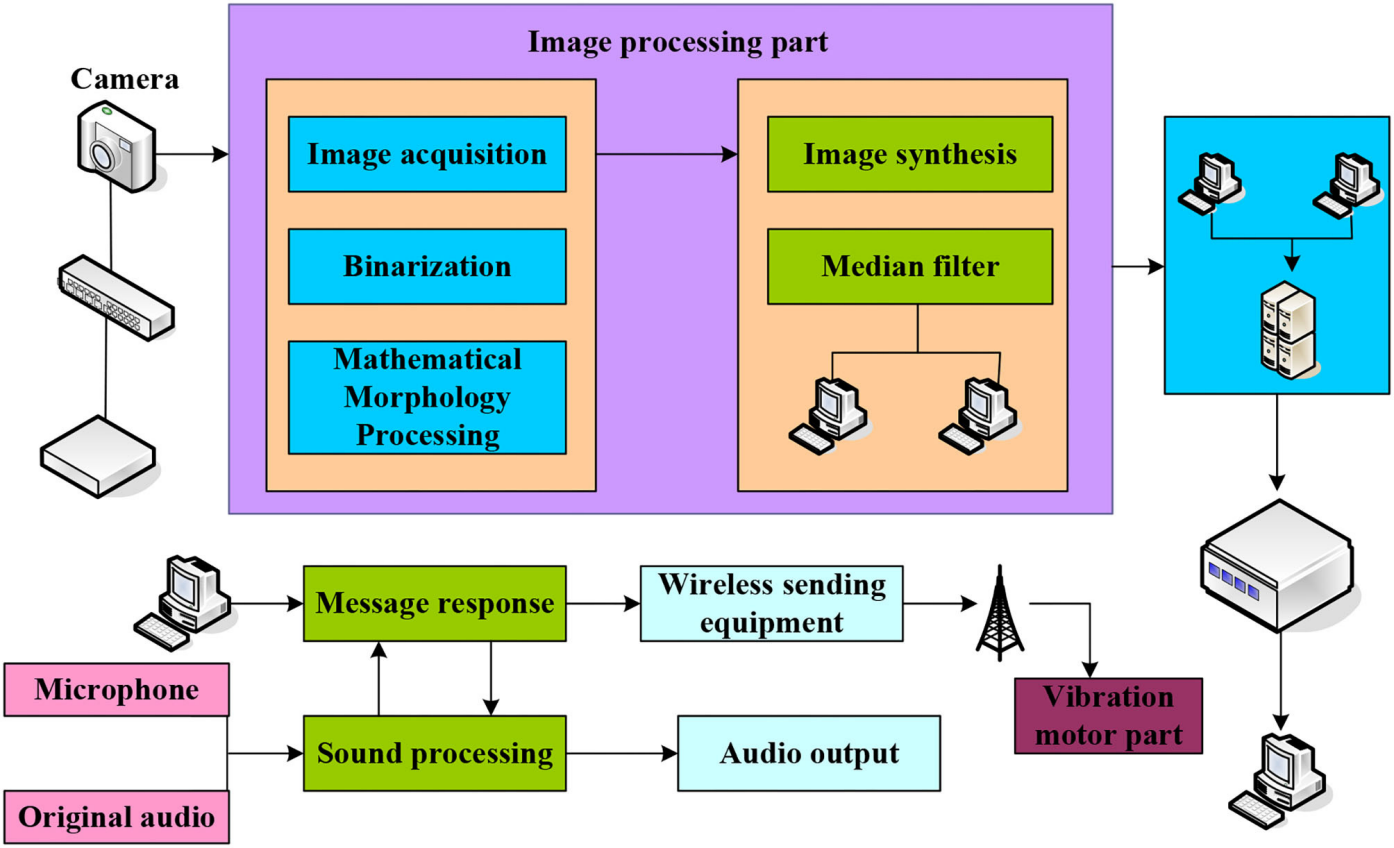
The overall structure design of the system.

The psychotherapy system we developed is equipped with three-dimensional music in virtual scenes. The processing unit in the system can virtualize people into the video, and realize the synthesis of prospects through VR and 3D modeling technology. The structure of the psychotherapy system is divided into three parts: vision, touch, and hearing. The wireless transmission technology used can improve. The overall structure design of the system is shown in Figure 1.

The support count is represented by δ(*x*), which is used to record the number of things included in the item set X (Burnell, 2018).


(9) $$\begin{array}{l} \delta \;(x)=|\left\{t_{i}|x\subseteq t_{i},t_{i}\in T\right\}| \end{array}$$

(10) $$\begin{array}{l} \text{s}=Q\left(\text{x}\right)=\frac{\delta \;(x\cup y)}{m} \end{array}$$

(11) $$\begin{array}{l} c\;(x)=\frac{\delta \;(x\cup y)}{\delta \;(x)} \end{array}$$

(12) $$\begin{array}{r} P_{R}\;(C=c_{i}|A_{1}=a_{1}\wedge \ldots \;A_{n}=a_{n})\\ =\frac{P_{R}\;(A_{1}=a_{1}\wedge \ldots \;A_{n}=a_{n}|C=c_{i})}{P_{R}\;(A_{1}=a_{1}\wedge \ldots \;A_{n}=a_{n})}P_{R}\;(C=c_{i}) \end{array}$$

The information gain rate is defined as follows (Sonuga-Barke et al., 2017):


(13) $$\begin{array}{l} GR\;(A)=\frac{G\;(A)}{S\;(A)} \end{array}$$

The split information is used in the above formula to standardize the information gain. The split information is similar to Info(D), which is defined as (Reardon et al., 2017):


(14) $$\begin{array}{l} SInfo\left(D\right)=-\overset{x}{\underset{j=1}{\sum }}\frac{D_{j}}{D_{i}}\times \ln\left(\left|\frac{D_{j}}{D_{i}}\right|\right) \end{array}$$

### Psychological Assessment Module

The functions that need to be realized in the psychological evaluation process include: college student users can view the list of psychological evaluation test questions, and select the psychological evaluation test questions for mental health evaluation. The system can save the results of the psychological evaluation during the process of opening the psychological evaluation or directly submit the results of the psychological evaluation to view an analysis of the results. The psychological assessment questions browsed by college students are untested and the results are valid. For the psychological test questions evaluated, they cannot participate in the psychological evaluation again, they can only view the psychological evaluation record from the psychological evaluation history.

(1) Test question bank management module

The system can modify, view, and recall test papers. The most popular, authoritative, and widely applicable test scales were added to the system. These include the Symptom Self-Rating Scale-SCL90, which is suitable for testing those that might have a psychological disorder, including what kind of psychological disorder and its severity. We also use the University Personality Inventory (UPI), through which students automatically classify the UPI results and perform statistics. Other scales used include the Selfrating Anxiety Scale (SAS) and Selfrating Depression Scale (SDS) test, which use computers to analyze, classify and count the collected psychological information. These scales are used in the automation, informatization, and computerized management of the students' mental health assessment system. The super administrator can perform maintenance, modifications, and view test papers.

(2) View test result module

The view function enables users to view their test results. After the system has finished scoring and given corresponding measures, users can click “View Test Results” to see their results and measures. Before the system generates a measure, the “view test result” is inoperable. The evaluation result table is used to save the test paper number of the test conducted by a single student and the evaluation result obtained through a fuzzy comprehensive evaluation. The fuzzy comprehensive evaluation method is a comprehensive evaluation method based on fuzzy mathematics. The comprehensive evaluation method transforms qualitative evaluation into quantitative evaluation according to the membership degree theory of fuzzy mathematics that is, using fuzzy mathematics to make a general evaluation of things or objects restricted by many factors. It connects with the student information table through the “study field,” and connects with the test paper table through the “examination paper number” field. The design of the evaluation result table is shown in Table 1.

**Table 1:** Design of evaluation result table.

**Field Name**	**Type of data**	**Length**	**Instruction**
Sy_ id	Varchar	10	Paper number
S_id	Varchar	20	Atudent ID
S_name	Varchar	10	Name
Result	Ntext	10	Evaluation result

### Online Consultation Module

Online counseling means that college students can communicate online through psychological counseling messages. When in the counseling center, they can click the counseling button to enter the online psychological counseling message. To avoid too frequent online comments, each time it submits a message, it needs an interval of 1 min before it can make the next message. At the same time, it can upload only one picture for each psychological consultation message submitted. At the same time, they can send a designated counselor a message or choose a counselor to remain a message. This will push the message to all psychologist clients.

The wrong substitution rate formula can be introduced (Manjunatha et al., 2017):


(15) $$\begin{array}{l} {\text{p}}_{x}=\frac{n-n^{\prime }}{m} \end{array}$$

n represents the number of samples on the branch (Koenen et al., 2017).

The classification error rate at node t is:


(16) $$\begin{array}{l} r\;(t)=e\;(t)/n\;(t) \end{array}$$

The data mining algorithm revised it to (Davis et al., 2018):


(17) $$\begin{array}{l} r\;(t)=\left[e\;(t)+1/2\right]/n\;(t) \end{array}$$

Then the classification error rate of *T*_*r*_ is (Felitti et al., 2019):


(18) $$\begin{array}{l} \text{r}\;(T_{r})=\frac{\underset{s}{\sum }\left[e\;(s)+1/2\right]}{\underset{s}{\sum }\left[n\;(s)\right]}=\frac{\underset{s}{\sum }e\;(s)+\frac{L\left(S\right)}{2}}{\underset{s}{\sum }\left[n\;(s)\right]} \end{array}$$

For the sake of simplicity, in quantitative analysis, the number of errors is used instead of the error rate, then (Kiirats, 2017):


(19) $$\begin{array}{l} e\;(t)=e^{\prime }\;(t)+\gamma \end{array}$$

For subtree *T*_*r*_, there are (Joshi and Prasad, 2019):


(20) $$\begin{array}{l} r\;(T_{r})=\left[e\;(T_{r})+1/2\right]/n\;(T_{r}) \end{array}$$

The standard error is defined as (Sana et al., 2018):


(21) $$\begin{array}{l} S\text{e}\sqrt{r\;(T_{r})}=\sqrt{\left[e\;(T_{r})+1/2\right]/n\;(T_{r})} \end{array}$$

### Design of Mental Health Early Warning Module

**Figure 2:**
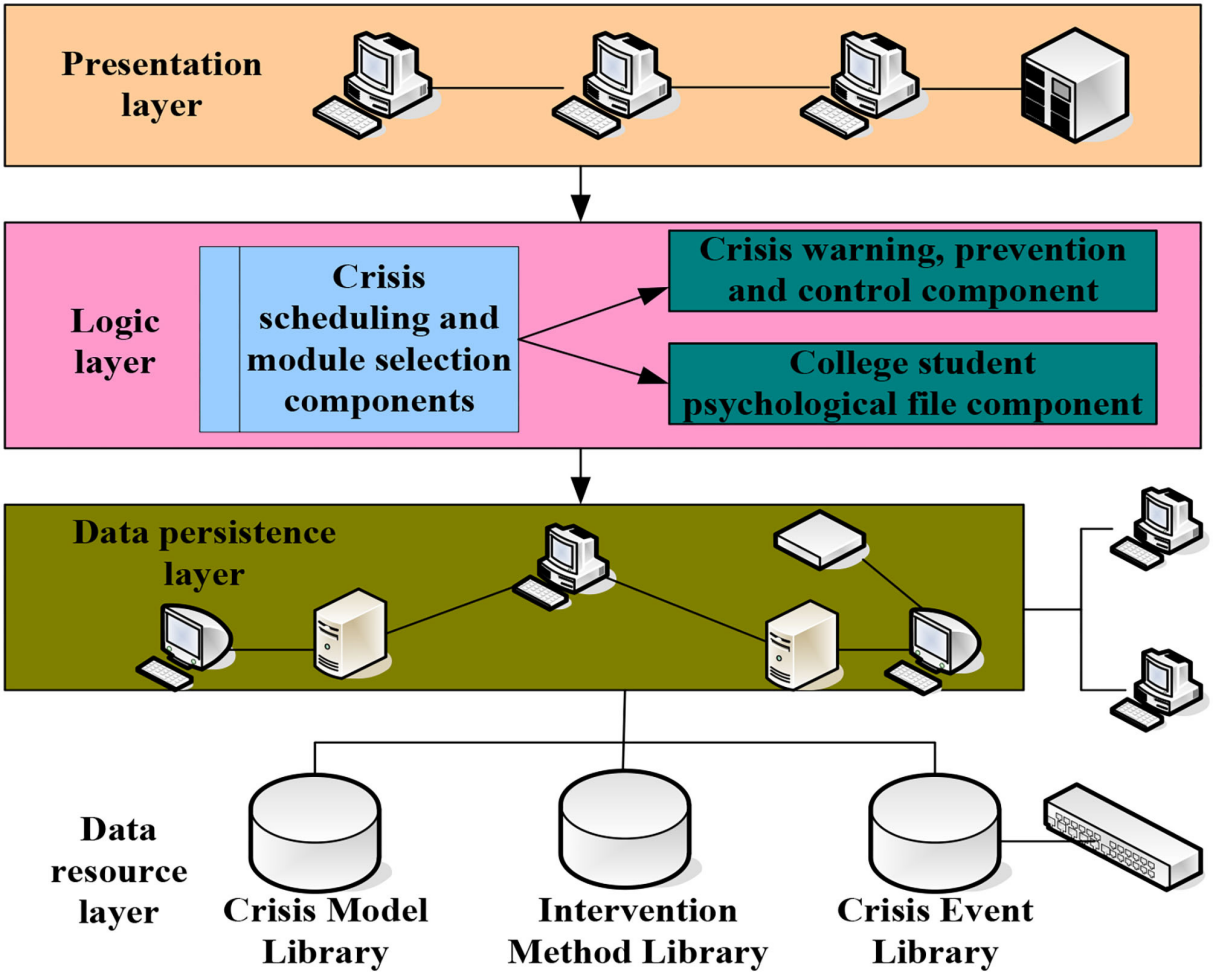
The logical structure of the early warning module.

Through the analysis of the main events of college student's daily life, with the help of professional psychological test scales, reasonable module selection is made. It uses the multi-agent system to simulate human decision-making methods to provide early warning, prevention, and control of the psychological crisis of vocational students. The process starts with crisis events that may induce daily study and life are divided into six major items and several sub-items (life events, academic events, interpersonal events, health, reward and punishment events, and environmental events). It then uses the auxiliary psychological scale measurement, according to the predetermined processing module, relying on the early warning and intervention model of the system from identification, measurement, analysis, prevention, and control, to evaluation. In this way, we will construct a psychological crisis prevention and control system for higher vocational students using network technology and multi-agent systems. The logical structure of the early warning module is shown in Figure 2.

Through the analysis of inductive crisis events, distinguishing individual differences among college students, and simulating human decision-making ideas, it completes effective information transfer, and then transmits the crisis information through the information exchange platform. Finally, it uses a multi-agent system to coordinate the relationship between members, reducing harm through timely psychological counseling and intervention. This article combines relevant theories of agents to establish a multi-agent. The following steps are taken:

1. It recognizes the crisis situation of the agent. It first recognizes the source of college students' psychological crisis and classifies it. Then understands the events and reasons that caused the psychological crisis and provides psychological counseling and intervention ideas based on the experience in the identification system.
2. It measures the characteristics of the agent's crisis. It analyzes their internal state (such as variables) and behavioral rules (such as functions, methods, etc.).
3. It establishes a multi-agent crisis early warning and intervention management system. It undertakes psychological crisis management and evaluation based on the established model and solves the problems of coordination between members of the system and their interaction (such as communication, negotiation, conflict handling), and so on. Through the analysis of the crisis events of college students, according to the module selection given in the previous section, a multi-agent system of college student crisis early warning and intervention model MASCM (Bastug et al., 2017) is proposed.

(22) $$\begin{array}{l} MAEBRCM=<AG,IFM,AT,DM> \end{array}$$

Here, according to the composition of the system, the crisis events are divided into life events, academic events, interpersonal events, health and loss, reward and punishment events, and environmental events (Avio et al., 2017).


(23) $$\begin{array}{l} IFM=\langle l_{1},l_{2},\ldots ,l_{n}\rangle \end{array}$$

Among them, *IFM* is the crisis category to which the i-th Agent belongs.

The selection principle of any agent for negotiation and decision-making is considered from the difference between the benefits obtained and the costs paid on the premise that it is in line with the overall interests of the school's student management (Lenoir et al., 2017):


(24) $$\begin{array}{l} U=\overset{n}{\underset{i=1}{\prod }}u \end{array}$$

(25) $$\begin{array}{l} U_{i}=R_{i}-C \end{array}$$

Among them, *U*_*i*_ is *A* the return obtained through measures such as crisis retention, crisis transfer, or crisis control.

## Mental Health Adjustment Results of College Students

**Figure 3:**
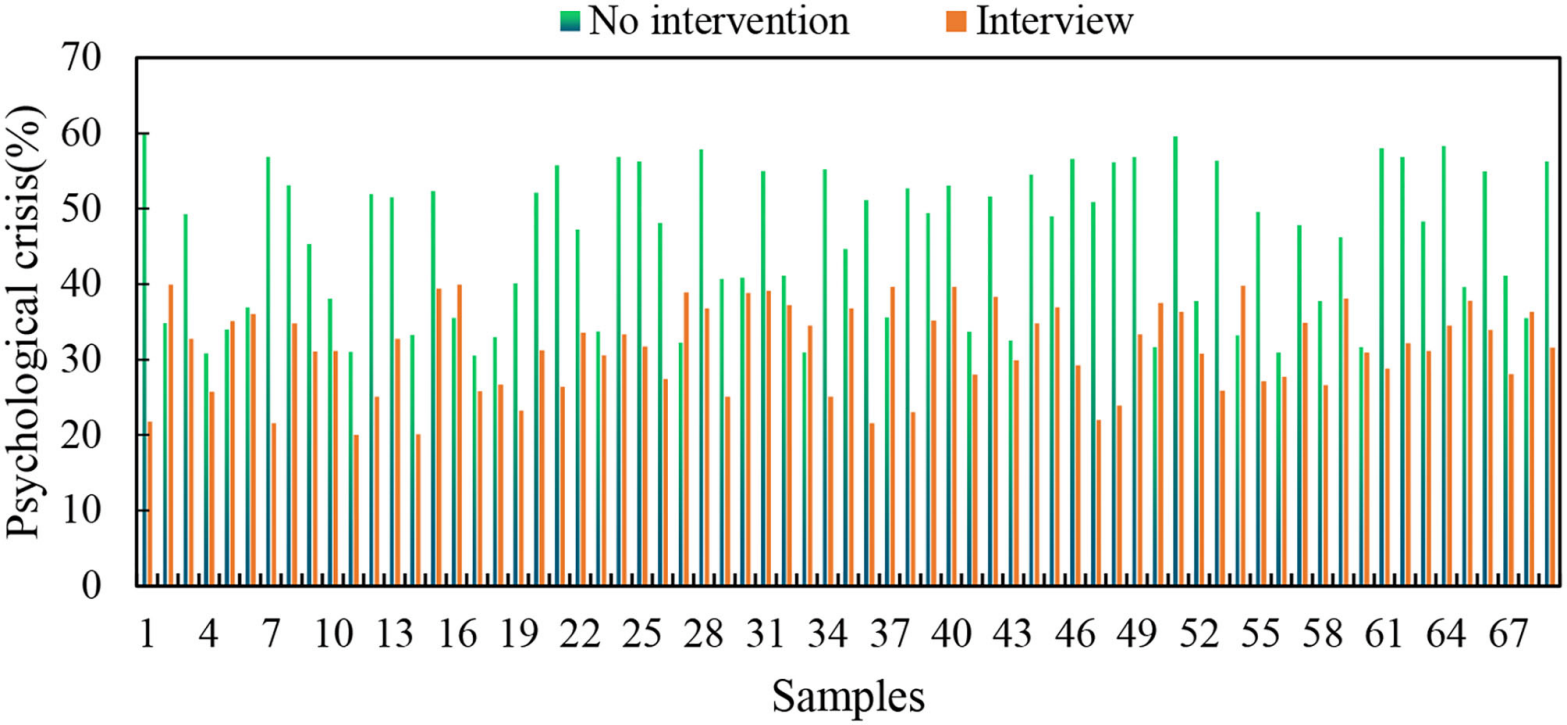
Psychological teacher interviews reduce the degree of a psychological crisis in students.

Increasing the number of psychological teacher interviews can reduce the degree of psychological crisis, but the degree of reduction of psychological crisis in interviews is not as large as the psychological crisis over time, which shows that the psychological teacher interview alone cannot solve all the problems. Psychological teacher interviews reduce the degree of a psychological crisis in students, as shown in Figure 3.

**Figure 4:**
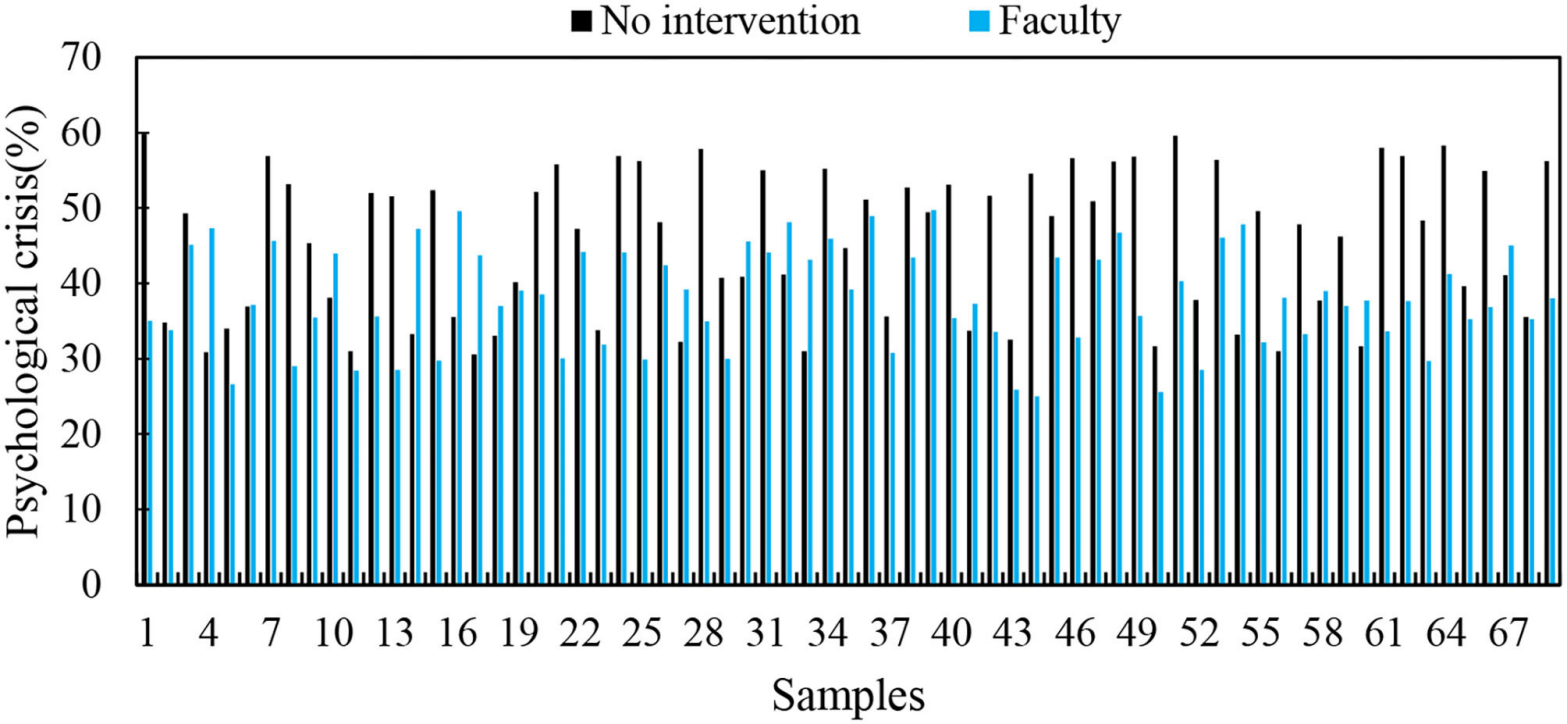
The impact of the improvement of the teaching quality of the teaching staff on the psychological crisis of students.

Since improvements for faculty and staff are relative, the influence of faculty and staff on the degree of psychological crisis is divided into two parts: anxiety and learning pressure. Therefore, the improvement of the quality of faculty and staff has a greater impact on the degree of psychological crisis, and the cumulative effect will effectively prevent the further deterioration of psychological crisis. Figure 4 shows the impact of the improvement of the quality of faculty on the psychological crisis of students.

The overall reliability of the Mental Health Literacy Scale for College Students is 0.799, and the Cronbach coefficient of each dimension is 0.695–0.860. The results show that the Mental Health Literacy Scale is good. Cronbach's alpha reliability evaluation is shown in Table 2.

**Table 2:** Cronbach's alpha reliability evaluation.

**Project**	**α coefficient**	**Number of items**
General questionnaire	0.799	22
Identify	0.695	6
Information	0.788	4
Belief	0.827	7
Manner	0.860	6

KMO test and Bartlett sphere test were performed on the first set of data (*N* = 380). The KMO value (the minimum standard is 0.70), and the associated probability of Bartlett's sphere test is 0.000. The test results of factor characteristic root, variance contribution rate, and variance cumulative contribution rate are shown in Table 3.

**Table 3:** Test results of factor characteristic roots, variance contribution rate, and variance cumulative contribution rate.

**Factor**	**Characteristic root**	**Cumulative contribution rate of variance (%)**
1	3.586	15.591
2	3.566	31.097
3	2.648	42.612
4	2.556	53.724

Because before the experiment will show the level of mental health literacy after the experiment, when verifying the intervention the pre-tested literacy score should be adjusted as a covariate. If the three application conditions of covariance analysis are met, covariance analysis is used. The results of covariance analysis are shown in Table 4.

**Table 4:** Covariance analysis results.

**Source**	**DF**	**Mean squared**
Revised model	3	1592.675
Intercept	1	1713.310
Grouping	2	223.024

**Figure 5:**
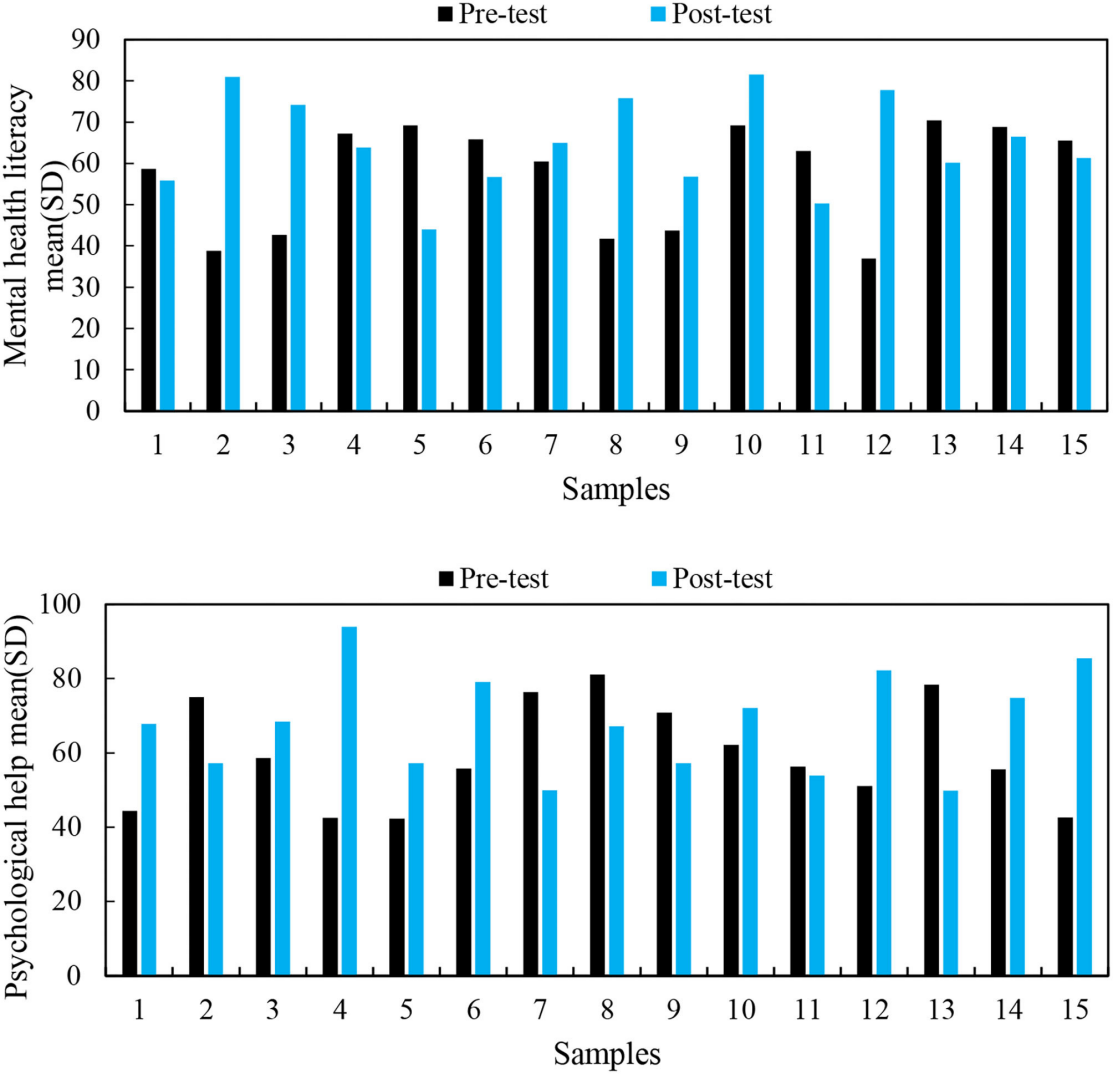
Mental health literacy and willingness to ask for professional psychological help of the experimental group and the control group.

First, it conducted a descriptive statistical analysis of the mental health literacy and the willingness to receive professional psychological help of the experimental group and the control group. The results are shown in Figure 5.

**Figure 6:**
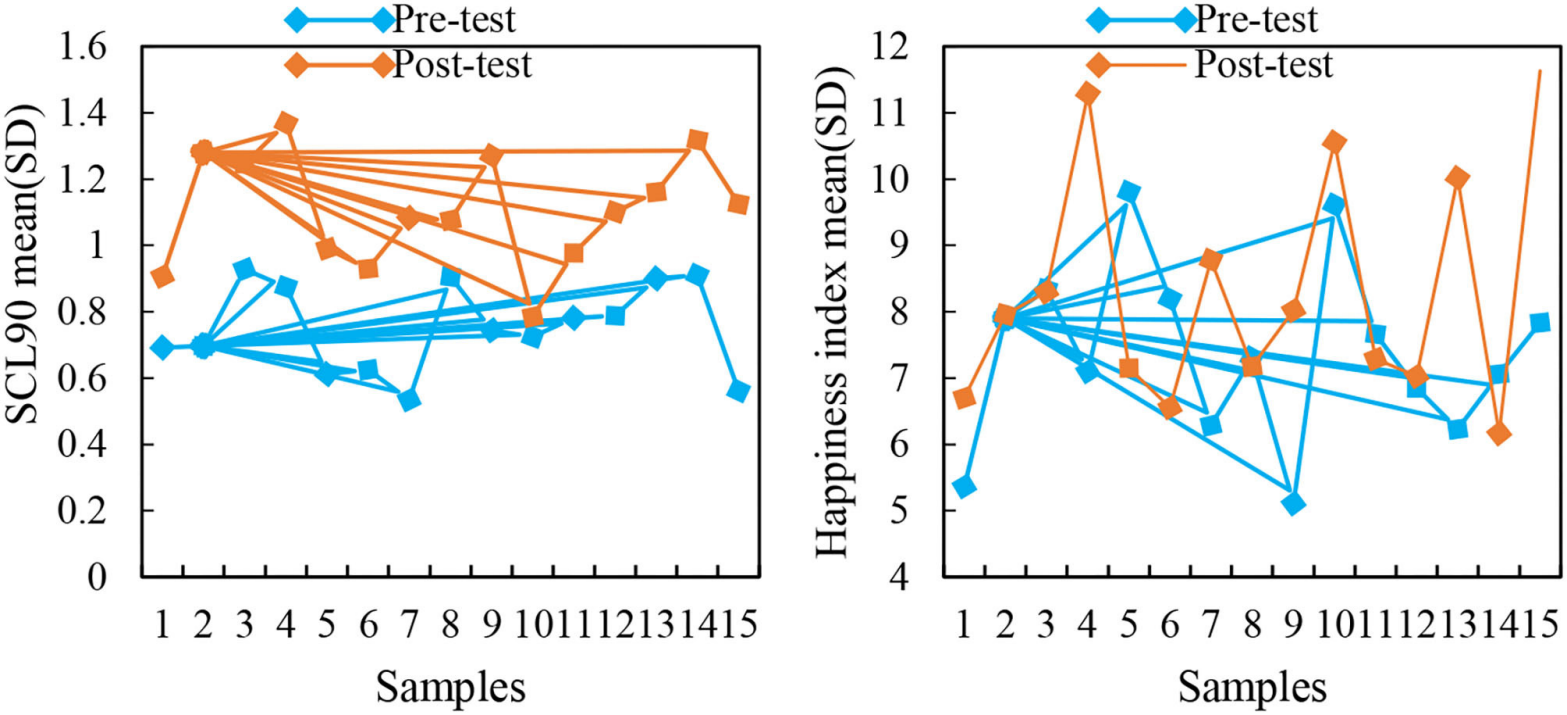
Before and after scores of SCL90 and happiness index.

The statistical results of the pre- and post-test scores of SCL90 and happiness index are shown in Figure 6.

The pre-test and post-test scores of the control group: *P* = 0.958 > 0.05. This shows that mental health literacy intervention has a significant effect on improving the willingness to ask for professional psychological help of college students in the experimental group. The test results of professional psychological help willingness samples are shown in Table 5.

**Table 5:** Sample test results of professional psychological willingness to ask for help.

**Group**	**Professional psychological help willingness score**	
	**Pre-test**	**Post-test**
	**Mean (SD)**	**Mean (SD)**
Test group	7.45 (1.94)	8.15 (1.76)
Control group	7.13 (2.15)	7.14 (2.21)
T(*P*-value)	0.89 (0.373)	2.86 (0.005)

Independent sample *t*-test and paired *t*-test were performed on the scores of the “SCL90” pre- and post-tests of the experimental group and the control group. SCL90 test results are shown in Table 6.

**Table 6:** SCL90 test results.

**Group**	**Professional psychological help willingness score**	
	**Pre-test**	**Post-test**
	**Mean (SD)**	**Mean (SD)**
Test group	1.41 (0.34)	1.27 (0.30)
Control group	1.54 (0.44)	1.46 (0.40)
T(*P*-value)	−1.97 (0.051)	−3.01 (0.003)

**Figure 7:**
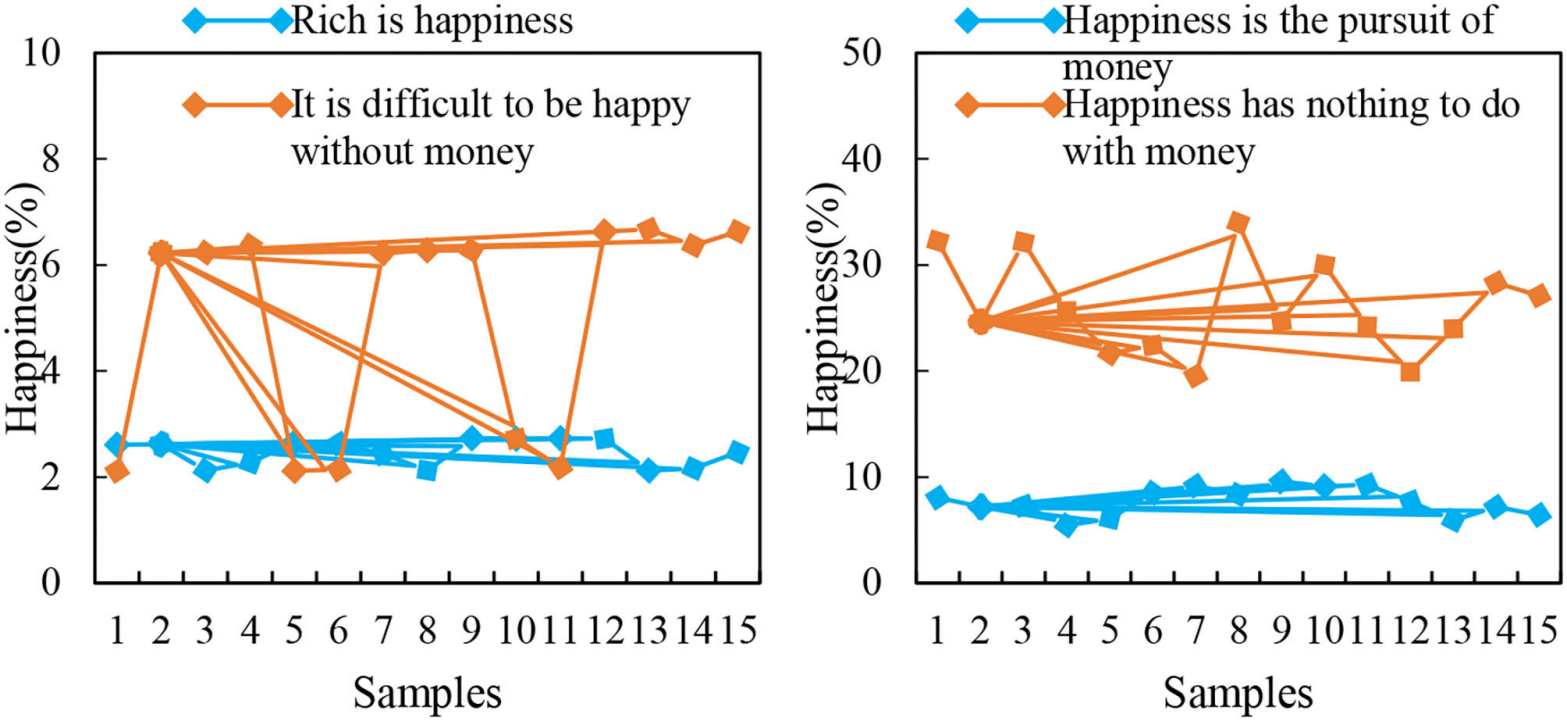
The relationship between happiness and money guided by positive psychology.

Analysis of the questionnaire indicates that the happiness of female students is generally positive and that they can more accurately understand the relationship between happiness and money, and the relationship between creating happiness and enjoying happiness. In the process of pursuing happiness, they have a firm will, are full of confidence about their future happiness, and believe that happiness comes from their hard work. However, there are also a few female college students who have certain misunderstandings in terms of acquisition methods and thoughts of happiness. This phenomenon should be given sufficient attention. The relationship between happiness and money under the guidance of positive psychology is shown in Figure 7.

**Figure 8:**
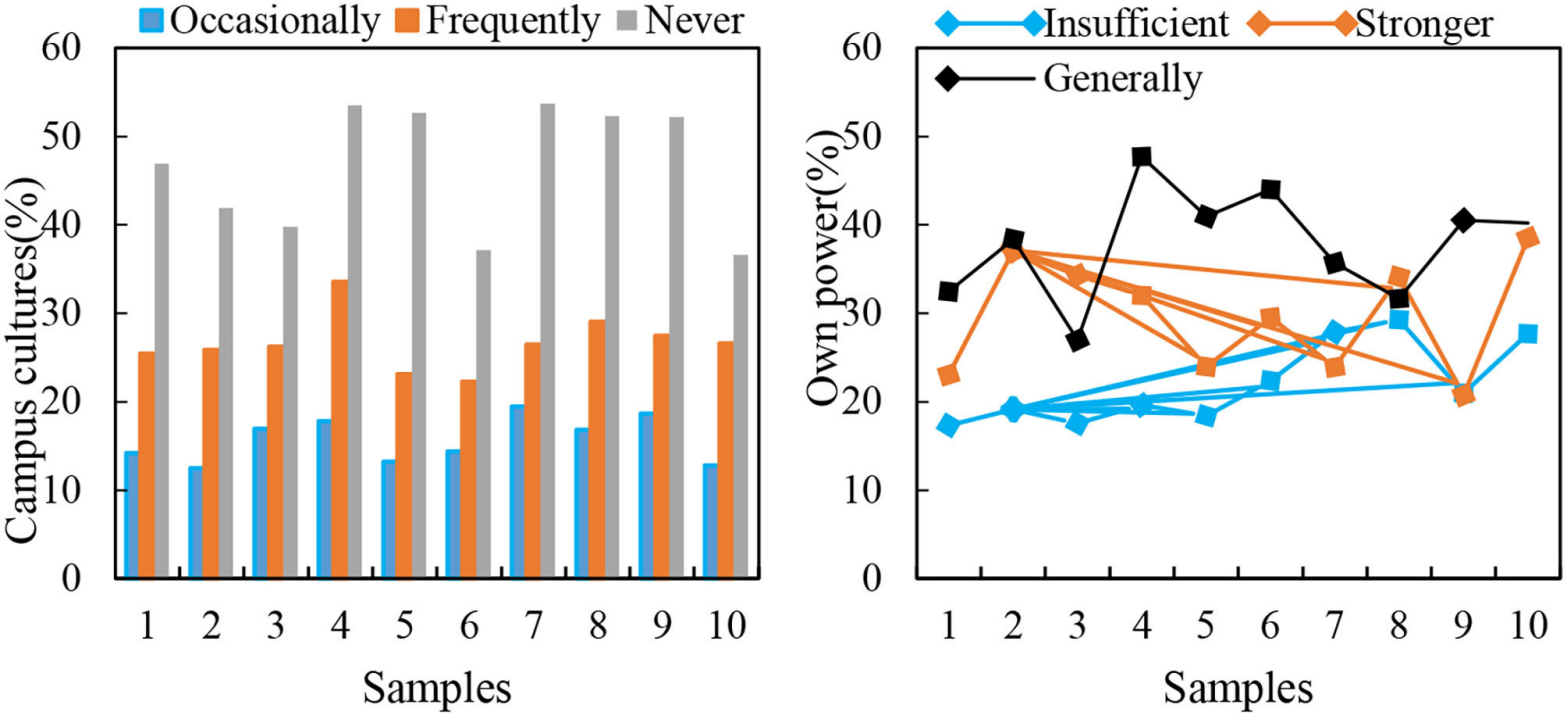
On abilities and participation in campus cultural activities.

With the serious phenomenon of education marketization and utilitarianism, some people regard the cultivating talents needed by society as the sole purpose of education. The school becomes the “factory,” and the students are the “products” produced. When answering “Do you often participate in campus cultural activities, and how do you feel?,” 11.54% of female students participated, outlining that campus cultural activities can provide exercise for people and are very meaningful. In total, 58.57% of female college students sometimes participate in campus cultural activities, depending on their interests. Finally, 29.88% of female students hardly participate in campus cultural activities and are not interested in such activities. Education is getting more and more deviated from students' happiness, meaning the balance between body and mind is neglected. Happiness education is rarely involved in the curriculum of colleges and universities. Although some schools take happiness courses as elective courses, they only instill the concept of “happiness” and lack practical activities. Moreover, there is no “student happiness-oriented” educational atmosphere, which makes some students' understanding of happiness simplistic and one-sided, meaning that some cannot discover, feel and create happiness. Figure 8 shows ability and participation in campus cultural activities.

**Figure 9:**
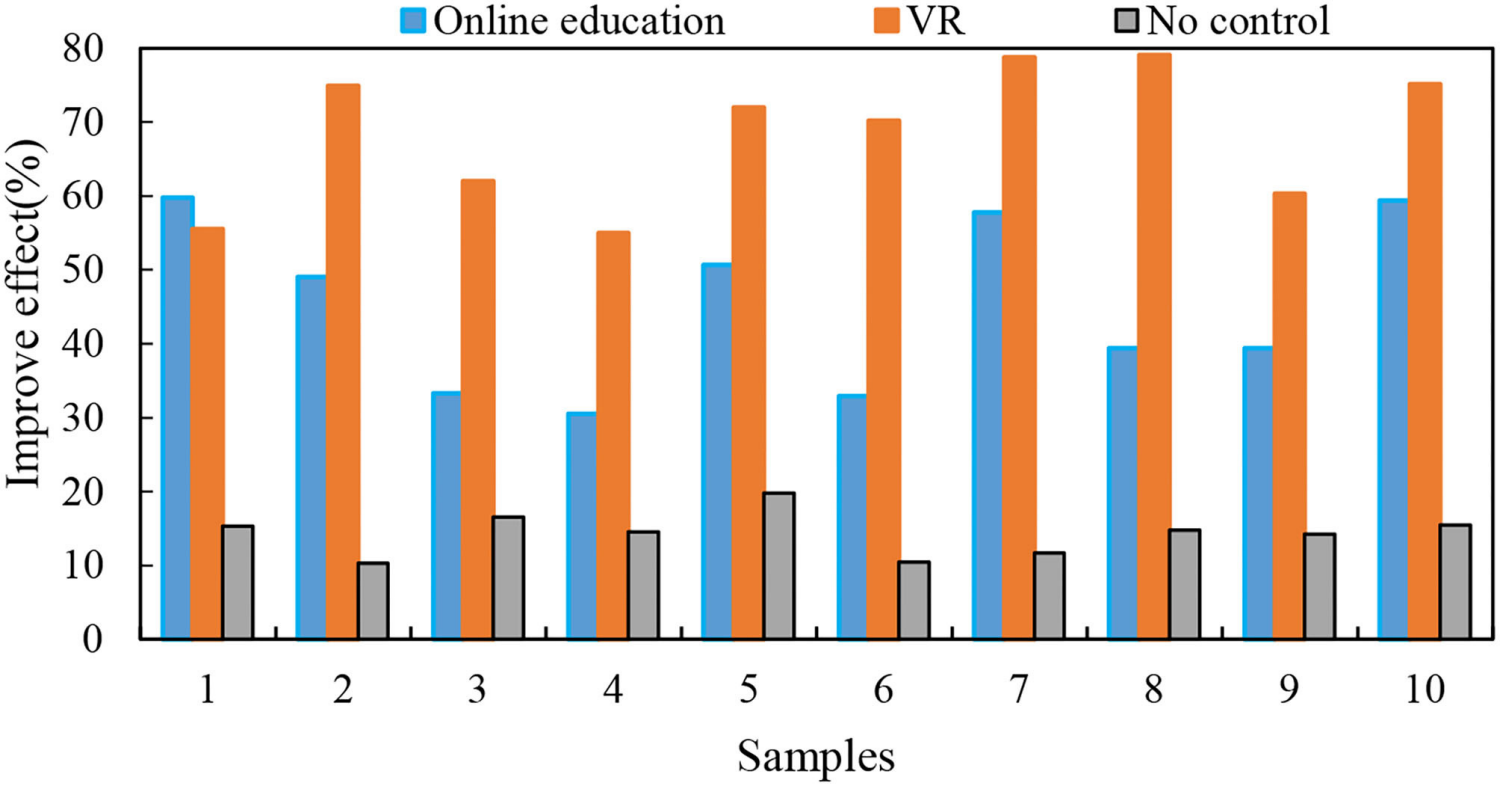
Virtual reality treatment system test results.

The post-test happiness index score of the virtual reality treatment system happiness index in the online education group was not significantly higher than the pre-test, *P* = 0.516 > 0.05. This shows that the program designed in this research could significantly improve the happiness of college students. The test result of the virtual reality treatment system is shown in Figure 9.

## Discussion

In recent years issues such as disease, environmental pollution, and mental health have become increasingly serious social problems. The knowledge that people need is becoming more and more complex. Only by constantly learning new knowledge can we cope with the increasingly fierce competition in today's society. College students have not yet fully integrated into society, but still have to face these social problems. Students have learning tasks, ideals, simple interpersonal relationships, and must think about other issues such as how to choose a career in the future and what mental states will enable them to face the complex social and other issues of life. Enabling them to control psychological impulses and actively adjust their mentality, means that they can optimistically accept and fully adapt to their social environment, overcoming psychological obstacles or mental illness. Using advanced psychological education concepts to improve the psychological teaching of college students enables them to formulate excellent mental health that conforms to national conditions (Kasza et al., 2017; Deepak et al., 2020). Providing college students with mental health education, teaching, and consulting work will facilitate the vigorous development of information technology and network technology and its rapid penetration into the field of education, the information source for college students to acquire mental health knowledge has rapidly shifted from teachers and libraries to a richer, flexible, and multi-media network. Undergraduate mental health educators must understand the network system architecture, working principles, transmission technology, and other information knowledge, which is beneficial to college students' mental health education.

The unbalanced development of various regions and industries, and the establishment of the social security system takes time and process. As a result, the gap in financial support for college students' families is gradually widening. Having good physical and mental health enables students to establish a positive awareness, understand puzzles, their studies, and life, and enhance their consciousness and initiative to adapt to society. Students need to learn to coordinate interpersonal relationships, continuously improve learning efficiency, enrich learning methods, and develop conscious potential. They also need to understand the standards of a sound personality (The healthy personality is the normal and harmonious development of the personality.) and have a basic grasp of unsound personalities (Imperfect personality refers to the long-term instability of self-awareness and self-image, and the existence of defects in one's own character.) and their manifestations, to actively improve their own personality. They need to understand the pros and cons of love, cultivate a healthy concept of love, understand career planning and career selection standards, and have a reasonable outlook on life, to fully recognize the reality of themselves, including their strengths and weaknesses, etc., enabling them to develop their strengths and skills, and identify and improve other areas of life.

## Conclusion

The social environment faced by college students is becoming more and more complicated, and the pressures of study and employment are increasing, meaning they have become a social group that is prone to problems. The cultivation of positive qualities is a key factor to improving the lives and contributions to society of students, and realizing their self-growth. This article has discussed mental health data, ways to obtain data, and some aspects to pay attention to when obtaining data. Positive mental health qualities also contribute to college, enhancing students' ability to self-learn, and promoting the improvement of academic performance. The article analyzed the feasibility of implementing networked education. After investigation, the goal of system construction was clarified, and the overall functional framework of the system was formed. Mental health consulting services are complex and provide good models. They also distinguished the different service requirements of users/clients and provide special functions according to the different service requirements of each individual. We developed a special service system that enables students to communicate with their teachers about their psychological problems and their teachers were able to respond to their questions and find out the causes through exploration, summarizing the results. The present study also suggests countermeasures to enhance the effect of problem responses.

## Data Availability Statement

The original contributions presented in the study are included in the article/supplementary material, further inquiries can be directed to the corresponding author.

## Author Contributions

All authors listed have made a substantial, direct, and intellectual contribution to the work and approved it for publication.

## Conflict of Interest

The authors declare that the research was conducted in the absence of any commercial or financial relationships that could be construed as a potential conflict of interest.

## Publisher's Note

All claims expressed in this article are solely those of the authors and do not necessarily represent those of their affiliated organizations, or those of the publisher, the editors and the reviewers. Any product that may be evaluated in this article, or claim that may be made by its manufacturer, is not guaranteed or endorsed by the publisher.
